# Factors Influencing Parental Decisions on Respiratory Syncytial Virus Immunoprophylaxis

**DOI:** 10.1016/j.jpedcp.2025.200153

**Published:** 2025-06-06

**Authors:** Katherine E. Shedlock, Steven D. Hicks, Ruth E. Gardner, Leah D. Kaye, Brody J. Lipsett, Eric W. Schaefer, Ian M. Paul, Benjamin N. Fogel

**Affiliations:** 1 Pediatrics, Penn State College of Medicine, Hershey, PA; 2 Public Health Sciences, Penn State College of Medicine, Hershey, PA

**Keywords:** vaccine, immunization, vaccination consent, vaccination hesitancy, trust, nirsevimab

## Abstract

**Objectives:**

New respiratory syncytial virus (RSV) immunizations for infants and pregnant mothers recently became available to prevent severe RSV disease in infants. We aimed to determine the primary reasons for parental RSV immunization decisions. We further sought to evaluate the associations between vaccine receipt and source of health care information and trust in one's health care provider.

**Study design:**

A convenience sample of parents and guardians of infants were surveyed during the 2023-2024 RSV season in one newborn nursery and three affiliated clinics that are part of an academic health system.

**Results:**

Among the 118 respondents, 79 (66.9%) chose to receive an RSV vaccine themselves (n = 42) or consented for infant immunization (n = 37). Thirty-nine (92.9%) parents who consented to maternal vaccination and 35 (87.5%) who consented to infant immunization stated a primary reason was protection for their infant. Among those that did not receive the maternal vaccine, the most common reasons were nonavailability (39.7%) or no provider immunization offer (22.2%). Infant immunoprophylaxis was most commonly refused due to the immunization being too new (66.7%). There were no significant associations between vaccine receipt and reported source of health information or between vaccine receipt and degree of trust in the health care provider.

**Conclusions:**

The desire to protect their infant from illness was the primary reason for parental RSV immunization intent, while the primary reasons for not immunizing were lack of availability, lack of provider recommendation, and the perception that the immunizations are too new. Ensuring availability and strong recommendations may improve immunization uptake.

## Highlights

• Most common reason for parental RSV immunization interest is infant protection.
• Provider recommendations for RSV immunizations are very influential.
• Parents are more concerned nirsevimab is too new compared to maternal RSV vaccine.

Respiratory syncytial virus (RSV) is the leading cause of acute lower respiratory tract illnesses (LRTIs) in infants, infecting almost every child before age 2.^1^, ^2^, ^3^ RSV infection can range from mild upper respiratory symptoms to severe respiratory illnesses, such as bronchiolitis, with hospitalization and respiratory failure, even among those without risk factors of prematurity or chronic lung disease.^3^

Prior to 2023, palivizumab, a monoclonal antibody, was the only approved RSV immunoprophylaxis, and it has been available only to those meeting specific high-risk criteria, such as prematurity, bronchopulmonary dysplasia, and congenital heart disease. In August 2023, two new advances in RSV prevention became available. A maternal RSV vaccine, RSV prefusion F protein–based vaccine, was approved by the US Food and Drug Administration as a single dose between 32 through 36 weeks of gestational age of pregnancy.^4^ Also, the Advisory Committee on Immunization Practices (ACIP) recommended a single dose of a new recombinant human monoclonal antibody, nirsevimab, for all infants <8 months born during or just before RSV season regardless of gestational age or identified risk factors.^5^ The ACIP also recommended immunization for infants and children aged 8 through 19 months who are at increased risk of severe RSV disease and entering their second RSV season.^5^ In order to protect infants from RSV-associated LRTI, the Centers for Disease Control and Prevention recommended that either the pregnant woman should receive the RSV prefusion F protein–based vaccine or their infant should receive nirsevimab. At the start of the 2023 RSV season in the US, it was estimated that universal RSV protection of all infants would reduce medically attended RSV LRTIs by 300 000, hospitalizations by 25 000, and health care costs by $612 million.^3^

Vaccine hesitancy has occurred with the introduction of new immunizations, including COVID-19 and human papillomavirus (HPV) vaccines, with parents citing the newness of the vaccines as a common reason for declining.^6^, ^7^, ^8^, ^9^, ^10^, ^11^ In addition, the pandemic increased overall parental risk perception for childhood vaccines and contributed to a decline in global childhood immunization rates.^12^ New immunizations in the postpandemic era are therefore subject to potentially high rates of refusal.

In order to tailor future public health efforts to improve RSV immunization uptake it is important to understand the reasons behind parents’ immunization choices. In this study, we asked caregivers during the 2023-2024 RSV season about their RSV immunization decisions and contributing reasons for these choices for themselves and their age-eligible children. We also asked their usual sources of pediatric health information and their trust in health care providers. We hypothesized that the most common reason for parents to choose the immunizations would be related to a recommendation from their provider and to protect their infant from RSV. We expected that the most common reasons for declining the immunizations would be related to the newness or lack of provider recommendation. We also hypothesized that those that obtained information about health care for their children from their provider and highly trusted their provider, would be more likely to consent to RSV immunization, while those that indicated they obtained information from social media or did not trust information from their health care provider would be less likely to consent to the immunization.

## Methods

### Ethics Approval

This study was approved by the Penn State Institutional Review Board (STUDY00023704). Verbal consent was obtained from all participating parents before completing the questionnaire.

### Participants and Design

This cross-sectional survey was conducted from November 27, 2023, through March 31, 2024, in the newborn nursery at a single children's hospital and three affiliated pediatric primary care clinics in Central Pennsylvania. Parents and guardians of infants in the newborn nursery and parents and guardians of infants less than 8 months old, seen for at least 1 well visit at one of the three primary care practices, were invited to participate in the survey. The study dates coincided with the dates the RSV immunization first became available until the end of RSV season. A quick response code linked to the survey was displayed on research flyers given to families during admission in the newborn nursery and upon arrival for each of the infant's well visits until the infant turned 8 months. Research assistants also sent emails to families after outpatient appointments to provide the survey link to families that had not yet participated. Informed consent and survey questions were provided in English, Spanish, and Nepali, as these are the most common languages spoken by this patient population. A $15 gift card was offered to parents and guardians for participating in the survey. The infant immunization was occasionally unavailable due to a national supply shortage in the first season of distribution, but research flyers were consistently handed out to assess intent to immunize.

The inclusion criteria were infant gestational age ≥35 weeks and infant age <8 months at the time of survey completion. We excluded families with infants 35 weeks of gestational age and with children >8 months old at the time of survey completion, those who did not complete at least 50% of the survey items, and duplicate surveys for parents who completed more than 1 survey.

### Data Collection

Demographic information including sex, race, ethnicity, parent education level, household income, health insurance type, and marital status was collected by self-report on the survey. The survey assessed biological mother receipt of the maternal RSV vaccine and infant receipt of the RSV immunization. Contingency questions asked “What contributed to the decision to [get/not get] the maternal immunization? (Check all that apply),” “If you decided to [have/not have] your child receive the RSV immunization, which of the following factors contributed to the decision? (Check all that apply),” and “What is the most important reason to you?” Answers choices were written in clear language using first person responses, such as “I didn't want my child to be hospitalized.” Review of the electronic medical record was used to confirm if the child received the RSV immunization and to screen for duplicate responses.

Participants not intending to have their infant immunized on the day of the encounter were asked if they planned to immunize their child later in the RSV season. Reasons contributing to and the most important reasons for intent to delay or intent not to immunize were queried.

Parents and guardians were also asked where they tended to obtain health information related to their child (child's doctor or health care provider, family members or friends, Google, health-directed website, social media, and other). The word “Google” was used as it is a well-known search engine and a common name when referring to a quick online search accessible to the general population. The phrase “health-directed website” was used to allow respondents to indicate sources dedicated to health specific information. Parents were also asked to what extent they trust information from their child's health care provider (always, usually, sometimes, rarely, or never). When “other” was chosen, responses were reviewed by the study team to assign responses to one of the multiple choice options when applicable.

### Study Outcomes

The primary outcome was immunization uptake, defined as a mother-infant dyad having either received the maternal vaccine (through self-reporting) or the infant immunization (confirmed through electronic health record review). The secondary outcome was intent to immunize, defined as immunization uptake or a dyad indicating that the only reason the immunization was not received was that it was not available.

### Sample Size and Statistical Analysis

A convenience sample of all 658 infants less than 8 months old who were seen for at least 1 well visit between November 27, 2023, and March 31, 2024, were invited to participate. In addition, all 344 infants with a newborn nursery admission between December 12, 2023, and March 31, 2024, were invited to participate. A total of 169 families participated in the survey representing a response rate of 16.9%. Of these, 51 responses were excluded (24 did not meet inclusion criteria, 17 were incomplete, and 10 were duplicate responses) leaving 118 responses that were included in the analysis.

Reasons for receipt and nonreceipt were reported descriptively. Association between sources of health care information and immunization uptake/intent to immunize and between trust in health care provider and immunization uptake/intent to immunize were tested using χ^2^ tests.

## Results

### Study Subjects

All 118 respondents in the included data set stated they are a biological parent of the child. Most participants were white, female, and had private insurance (Table I). About two-thirds had a degree beyond high school. About half had a household income less than $100 000 per year.

**Table I:** Characteristics of participants

Characteristic	N = 118∗
Race/ethnicity of adults, n (%)	
Non-Hispanic White	94 (81.7)
Other	21 (18.3)
Gender of adult is female, n (%)	106 (89.8)
Education of adult, n (%)	
Some high school or high school graduate	39 (33.1)
College, trade school, or advanced degree graduate	79 (66.9)
Household income, n (%)	
<$100 000	55 (48.2)
≥$100 000	59 (51.8)
Insurance type of adult, n (%)	
Private	79 (67.5)
Medicaid or self-pay	38 (32.5)
Marital status is married, n (%)	92 (78.0)
Sex of child is male, n (%)	67 (56.8)
Child attending out-of-home childcare, n (%)	40 (33.9)
Gestational age of child, weeks, mean (SD)	38.8 (1.2)
Birth weight of child, kg, mean (SD)	3.34 (0.5)

∗ Where responses do not add to 118 the remainder represents missing data.

**Figure 1:**
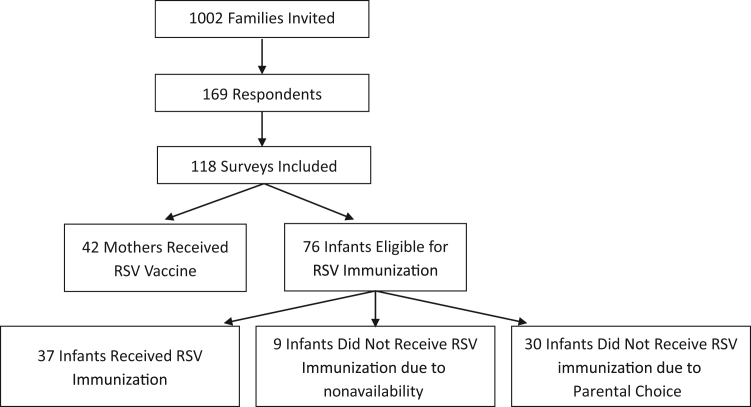
Flow diagram demonstrating response rate, survey completion rate, immunization intent, and immunization uptake. *RSV*, respiratory syncytial virus.

Out of the 118 dyads, 79 (66.9%) received either the infant or maternal immunization, 42 (35.6%) received the maternal vaccine, and 37 (48.7% of the 76 remaining dyads) received the infant immunization. When including respondents who would have consented to infant immunization if it were available, 88 (74.6% of 118 dyads) intended to receive RSV protection (Figure 1).

### Reasons for Vaccine Acceptance and Refusal

**Figure 2:**
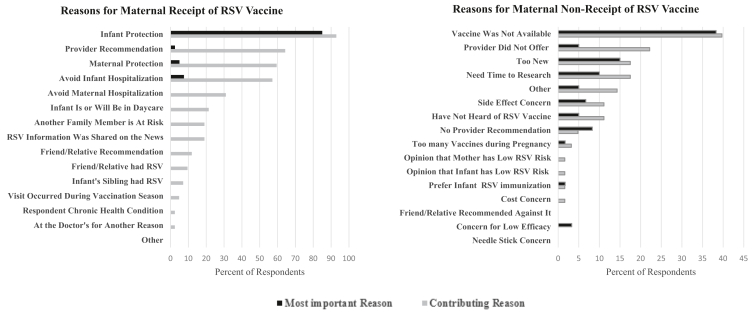
Self-reported most important and contributing reasons for parental decisions to receive or not receive the maternal RSV vaccine. n = 42 and 63, respectively.

The most commonly reported contributing factors for receiving the maternal RSV vaccine were wanting the infant to be protected (92.9%), recommendation from provider (64.3%), wanting maternal protection during pregnancy (59.5%), and wanting to avoid hospitalization for the infant (57.1%) (Figure 2). The majority of respondents (85%) indicated the most important reason was wanting protection for the infant.

In contrast, reasons for not receiving the maternal RSV vaccine were vaccine nonavailability (39.7%), not offered by the provider (22.2%), and too new of a vaccine (17.5%) (Figure 2). The most important reason for 38.3% of respondents was that it was not available with other responses chosen infrequently.

**Figure 3:**
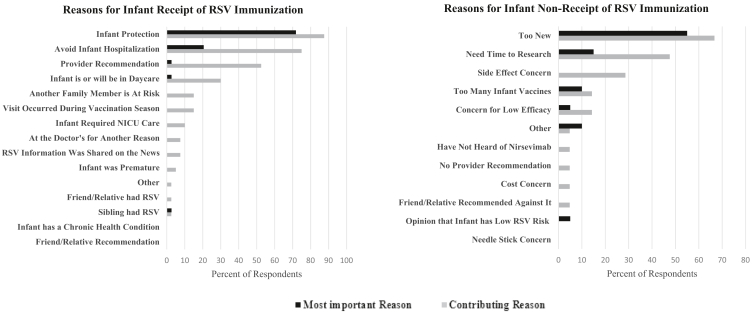
Self-reported most important and contributing reasons for parental decisions to consent to or decline the infant RSV immunization. n = 40 and 21, respectively.

Regarding infant RSV immunization, parents reported wanting the infant to be protected (87.5%), wanting to avoid hospitalization for the infant (75.0%), and recommendation from the provider (52.5%) as important reasons (Figure 3). The most important reason for 71.8% of respondents was protection for the infant, and the most important reason for 20.5% was avoiding hospitalization for the infant.

For those refusing the infant RSV vaccine, reasons included too new of a vaccine (66.7%), lack of time for enough personal research (47.6%), and worry about side effects (28.6%) (Figure 3). The most important reason for 55.0% of respondents was that the immunization was too new. For those respondents who stated they intended to have their child receive the RSV immunization later in the season, the most frequently reported reason for the delay was that the vaccine was not available (66.7%) with less frequent responses related to wanting more time to do personal research (26.7%) or feeling that there were already too many vaccines (6.7%).

### Sources of Health Care Information

The majority of respondents (94.9%) stated they receive health information from their child's doctor or health care provider. Fewer parents indicated that they receive health information from health-directed websites (33.9%), family members or friends (23.7%), Google (13.6%), or social media (5.1%). There were no significant associations between immunization uptake and information source. Results are displayed in Table II.

**Table II:** Association of immunization uptake with parental sources of health care information

Information sources n = 118	Immunization uptake [95% CI]	*P* value
Health care provider		.40
Yes	76/112 (67.9%) [58.3%-76.3%]	
No	3/6 (50.0%) [11.8%-88.2%]	
Family or friends		.42
Yes	17/28 (60.7%) [40.6%-78.5%]	
No	62/90 (68.9%) [58.3%-78.2%]	
Google		.68
Yes	10/16 (62.5%) [35.4%-84.8%]	
No	69/102 (67.6%) [57.7%-76.6%]	
Health-directed websites		.31
Yes	23/40 (57.5%) [40.9%-73.0%]	
No	56/78 (71.8%) [60.4%-81.4%]	
Social media		.09
Yes	2/6 (33.3%) [4.3%-77.7%]	
No	77/112 (68.8%) [59.3%-77.2%]	

### Trust in the Health Care Provider

A total of 53.4% of respondents indicated that they “always” trust information from their child's health care provider and 46.6% stated that they either “usually” or “sometimes” trust information from their child's health care provider. Only 5 respondents chose “sometimes” and no respondents reported “rarely” or “never.” Of those that “always” trust information from their child's health care provider, 71.4% received RSV immunoprophylaxis and of those that “usually” or “sometimes” trust information from their child's health care provider, 61.8% received RSV immunoprophylaxis (*P* = .27).

## Discussion

The most important and most common reason contributing to the parent's decision to receive an RSV immunization is the desire to protect their infant. Provider recommendations are also a strong influence. Common reasons for maternal nonimmunization are nonavailability and not being offered the vaccine, while the most common reason for infant immunization refusal is newness. There was no significant association between RSV vaccine uptake and reported health information source or between vaccine uptake and degree of trust in the child's health care provider.

Initial parental perceptions of the COVID-19 and HPV vaccine were similar to the results in this study, with parental hesitancy based on the vaccines being new. Acceptance of the COVID-19 vaccine was mixed, even during the height of the global pandemic.^7^ Notably, parents have been more likely to immunize themselves than their children, perhaps due to safety concerns.^7^^,^^8^ In general, parents that are well-informed about the safety profile of the COVID-19 vaccine experience less anxiety about the vaccine and are more likely to consent for their children.^7^ When the HPV vaccine became available, common caregiver stated reasons for vaccine refusal included lack of provider recommendation, concerns about cost, and newness of the vaccine.^9^ Over time, the rationale for refusal shifted to safety concerns and the lack of school entry requirements.^9^^,^^10^ HPV vaccine uptake has been shown to be associated with parental familiarity with the vaccine, positive perception of effectiveness, older age of the child, and a strong recommendation from a health care provider.^10^^,^^11^

It is encouraging that the most important and most common reason contributing to lack of maternal RSV uptake in the first year after introduction was immunization nonavailability and the second was not being offered the vaccine. This implies that as availability increases and obstetric providers consistently offer and recommend this vaccine when patients are eligible, rates of protection will increase substantially. Only 17.5% of the respondents that did not receive the maternal vaccine indicated they felt the vaccine was too new and that this contributed to their decision to decline it. Interestingly, the adult RSV vaccine was US Food and Drug Administration–approved and ACIP-recommended in the same year as nirsevimab, but families felt much more deterred by the “newness” of the infant RSV immunization, with two-thirds of respondents who declined it indicating that this was a reason that contributed to their decision. Parents can feel a high degree of anxiety with vaccine decisions, especially newer vaccines and the importance of physician counseling has been demonstrated in previous studies with often more time needed for counseling on newer vaccines.^13^, ^14^, ^15^ RSV immunization rates may increase as RSV protection becomes a more long-standing addition to routine schedules and no longer perceived as new.

Hinderstein et al utilized parental interviews during the first season of RSV immunization administration to determine common themes for infant RSV immunization decisions and demonstrated that major knowledge gaps about RSV prophylaxis exist, parents often feel they need more time to decide, and trust in the pediatrician improved parents’ perception of the immunization.^16^ These themes are consistent with our findings of the importance of a provider recommendation and that hesitancy can be related to a feeling of “newness.” Parental exposure to RSV prophylaxis information prior to the birth of the infant may be impactful.^16^ In order to improve knowledge gaps and reduce parental concern, RSV immunization discussions with families should emphasize their efficacy and safety. During the 2023-2024 RSV season, nirsevimab was 89% effective against medically attended RSV-associated acute respiratory infections and 93% against RSV-associated hospitalization^17^ with no increased incidence of serious adverse events for those that received the immunization.^5^

There was an initial concern in the health care community that there could be poor uptake of the infant immunization based on occasional parental concerns of too many vaccines being given already in this age group,^18^^,^^19^ but in our study, this was rarely chosen as a reason for declining the infant immunization. Further research could stratify by age of infant and encounter type to evaluate if parents are more likely to consent at encounters without other interventions.

With the pervasive use of social media, families are exposed to abundant accurate and inaccurate health care information.^20^ Social media can rapidly propagate vivid misinformation. A 2017 study examining 87 videos on the YouTube platform using the words “vaccine safety” and “vaccines and children” demonstrated that 65% of the videos expressed an antivaccine sentiment and 36.8% lacked any scientific evidence.^21^ Studies have demonstrated that adults that use social media as a source of vaccine information without any other trusted source are twice as likely to be vaccine hesitant,^22^ and parents of children that have nonmedical vaccine exemptions for school are more likely to obtain vaccine information from sources other than health care professionals compared to parents of vaccinated children.^19^ In this study, there was no significant association between vaccine receipt and reported source of health information. It is reassuring that 95% of respondents list their health care provider as an important source of health care information, and relatively few (5%) respondents report social media as a source of their health care information.

Vaccine hesitancy is a leading global health threat and is influenced by trust in the effectiveness and safety of vaccines as well as in the system that delivers them.^20^^,^^23^ In this study, there is no significant difference in vaccine uptake between respondents who always trust information from their health care provider and those who usually or sometimes trust this information. It is reassuring that 96% of respondents indicated that they either always or usually trust information from their health care provider. No respondents chose that they “never” trust information from their health care provider; however, six parents did not choose “health care provider” as one of their answer choices for where they tend to receive health care information for their child. Some parents may consider other sources of health care information more important but still have a degree of trust in their health care provider.

### Limitations

This study was limited by its design as a single center and a convenience sample with small response rate and sample size. The majority of patients were privately insured, which limits generalizability. There is the possibility of sampling bias. Parents that were able to and desired to attend visits were more frequently invited to the study while those that may not trust the medical system, or were not able to attend due to socioeconomic barriers, were less likely to receive the invitation to participate. Sampling bias, as well as social desirability bias, may have contributed to our very low rates of reported mistrust of health care providers and use of social media as a source of health care information. Sampling bias was mitigated by inviting participants in multiple settings (ie, newborn nursery and outpatient clinic). Social desirability bias was diminished by using a confidential online survey separate from the patient encounter. Nonresponse bias may have allowed for the overrepresentation of families that favor vaccination. We attempted to limit this by offering all respondents a participation incentive. While exploring associations between outcomes and sociodemographic characteristics would have been informative, we did not have the sample size to be able to complete these analyses.

Further, provider recommendation has been shown in prior studies of new vaccines to be the most important influencer for vaccine uptake, but because of shortages in the first year the infant immunization was occasionally not available during the study dates and providers were not always able to offer it. Survey answers regarding nonavailability and health care providers not recommending or offering the immunizations are likely to have a higher percentage in our study than what would take place with consistent availability. Additionally, receipt of the maternal vaccine was self-reported and not confirmed in the medical record.

## Conclusions

We found that the primary driver for intent to immunize against RSV was the desire to protect the child from illness (effectiveness), while the primary influences for not immunizing were lack of availability, lack of provider recommendation, and immunoprophylaxis seeming too new. Parents commonly indicated concern for the infant immunization being too new, while they rarely indicated this concern for the maternal vaccine. We also found a high degree of trust in health care providers in this sample. These findings underscore what has been seen with other vaccines; a strong provider recommendation highlighting the effectiveness of disease prevention is critical to ensuring immunization uptake.^7^^,^^10^^,^^11^

## CRediT authorship contribution statement

**Katherine E. Shedlock:** Writing – review & editing, Writing – original draft, Visualization, Validation, Supervision, Resources, Project administration, Methodology, Investigation, Formal analysis, Data curation, Conceptualization. **Steven D. Hicks:** Writing – review & editing, Visualization, Validation, Supervision, Resources, Project administration, Methodology, Investigation, Formal analysis, Data curation, Conceptualization. **Ruth E. Gardner:** Writing – review & editing, Visualization, Resources, Project administration, Methodology, Investigation, Data curation, Conceptualization. **Leah D. Kaye:** Writing – review & editing, Visualization, Project administration, Methodology, Investigation, Data curation, Conceptualization. **Brody J. Lipsett:** Writing – review & editing, Visualization, Resources, Project administration, Investigation, Data curation, Conceptualization. **Eric W. Schaefer:** Writing – review & editing, Visualization, Validation, Software, Methodology, Investigation, Formal analysis, Data curation, Conceptualization. **Ian M. Paul:** Writing – review & editing, Visualization, Supervision, Software, Project administration, Methodology, Investigation, Funding acquisition, Formal analysis, Data curation, Conceptualization. **Benjamin N. Fogel:** Writing – review & editing, Visualization, Validation, Supervision, Software, Resources, Project administration, Methodology, Investigation, Funding acquisition, Formal analysis, Data curation, Conceptualization.

## Declaration of Competing Interest

This research was supported by the Ashley Nicole Shellenberger SIDS Research Fund at the 10.13039/100011594Penn State College of Medicine.
